# Navigating pelvic anatomy for exenteration: a clinical guide for radiologists

**DOI:** 10.1093/bjr/tqag136

**Published:** 2026-06-03

**Authors:** Stephanie Nougaret, Verity Wood, Phillipe Rouanet, Arend G J Aalbers, Tamara Glyn, Damian Tolan, Doenja M J Lambregts

**Affiliations:** Department of Radiology, Montpellier Cancer Institute, 34000 Montpellier, France; PINKCC Lab, U1194, Montpellier Research Cancer Institute, University of Montpellier, 34000 Montpellier, France; Department of Radiology, Health New Zealand Te Whatu Ora Waitaha, Christchurch Hospital, Christchurch, Canterbury, 8011, New Zealand; Department of Oncological Surgery, Montpellier Cancer Institute, 34000 Montpellier, France; Surgical Oncology, Netherlands Cancer Institute, Amsterdam, The Netherlands; Department of Surgery, Waitemata District Health Board, Auckland, New Zealand; Department of Radiology, Leeds Teaching Hospitals NHS Trust, Leeds, United Kingdom; Department of Radiology, The Netherlands Cancer Institute, Amsterdam, The Netherlands; GROW Research Institute for Oncology and Reproduction—University of Maastricht, 6200 MD, Maastricht, The Netherlands

**Keywords:** pelvic exenteration, pelvic anatomy, magnetic resonance imaging, neoplasm recurrence, local, preoperative planning, radiology, interventional/methods

## Abstract

Pelvic exenteration (PE) is a complex, potentially curative option for locally advanced or recurrent pelvic malignancies in which achieving an R0 margin is paramount. This clinical review provides a pragmatic, anatomy-driven framework to optimize preoperative assessment and multidisciplinary planning. Using a compartment-based schema—central, anterior, posterior, and lateral—the article maps tumor spread to structures that determine technical feasibility and functional outcomes. For each compartment, surgical implications and reporting “pearls and pitfalls” are summarized to ensure radiological interpretation directly informs surgical decision-making. Special attention is given to differentiating tumor infiltration from post-treatment fibrosis, particularly following chemoradiotherapy, where diffusion-weighted magnetic resonance imaging plays an important role in the attempt to differentiate residual disease from fibrosis and defining safe resection planes. The review outlines assessment of critical anatomical structures or variants that have implications for surgical planning or functional consequences for patients. In addition, reconstructive options and their imaging appearances are briefly discussed to aid postoperative assessment. Through detailed anatomical analysis and structured, compartment-based reporting, radiologists can enhance multidisciplinary communication, refine patient selection, anticipate morbidity, and ultimately increase the likelihood of achieving complete resection in pelvic exenteration.

## Introduction

Pelvic exenteration (PE) represents one of the most radical and technically demanding surgical procedures for the management of locally advanced or recurrent malignancies.^1^ Originally pioneered for recurrent cervical cancer, PE has evolved significantly and is now performed for a wide range of malignancies, including colorectal, gynecologic, urologic, and rare sarcomatous tumors.^2–5^ The primary goal of surgery is en bloc resection of all involved structures to obtain a complete (R0) resection, which remains the most important prognostic factor for long-term survival and local disease control.^6–9^

Unlike conventional oncologic resections, PE requires a comprehensive understanding of pelvic anatomy beyond classical embryologic and surgical planes.^10^ Tumor infiltration across compartments, previous treatments (radiotherapy, chemotherapy, surgery), and the resulting fibrosis and altered anatomy may obliterate normal surgical landmarks.^8^^,^^9^ As a result, PE frequently necessitates resection of multiple organs, bones, vessels, and nerves, challenging even for the most experienced surgical teams.^8^^,^^9^

Preoperative imaging has become an essential component of multidisciplinary planning for PE.^11–13^ Magnetic Resonance Imaging (MRI) remains the cornerstone for detailed local staging, while Computed Tomography (CT) and Positron Emission Tomography/CT (PET/CT) are complementary for assessment of lymphatic spread and distant staging.^11^^,^^12^^,^^14–16^ The recent multidisciplinary consensus guidelines from the European Society of Gastrointestinal and Abdominal Radiology (ESGAR), the Society of Abdominal Radiology (SAR), the European Society of Urogenital Radiology (ESUR), and the PelvEx Collaborative group have emphasized the central role of imaging in patient selection, surgical planning, and assessment of technical feasibility for radical resection.^17^

The ESGAR-SAR-ESUR-PelvEx guidelines highlight that beyond simply detecting tumor presence, imaging must enable precise mapping of tumor and fibrosis extent relative to critical anatomical structures such as bones, major vessels, pelvic nerves, ureters, and fascial planes.^17^ Structured reporting and compartment-based analysis are strongly encouraged to facilitate effective communication between radiologists and surgeons and to support decision-making at multidisciplinary tumor boards.^18–22^ Moreover, detailed preoperative knowledge of anatomical involvement directly informs surgical eligibility and strategies, anticipated morbidity, and functional outcomes.^17^

Despite the central role of imaging in PE planning, many reports still lack sufficient anatomical detail, and gaps remain in linking radiologic anatomy to key surgical decisions. Radiologists must move beyond organ-based assessment toward a compartmental understanding of pelvic anatomy, spatial relationships, and potential planes of dissection, providing reports that serve as true surgical roadmaps. Familiarity with both normal and post-treatment anatomy is therefore essential.

This review offers a structured, clinically oriented overview of pelvic organs, fascial planes, and anatomical spaces relevant to PE, aiming to equip radiologists with the anatomic insight required for optimal patient selection, precise surgical planning, and effective multidisciplinary communication.

## Protocol considerations

For a complete overview of recommendations for image acquisition in PE, we refer to the ESGAR-SAR-ESUR-PelvEx guidelines.^17^ In short, preoperative imaging should include MRI of the pelvis, complemented by CT of the chest, abdomen and pelvis (portal venous phase) and FDG-PET/CT from skull-base to thigh. The recommended MRI protocol includes high resolution T2-weighted sequences including sagittal and oblique views angled perpendicular to the tumor axis and high b-value (b800-1000) diffusion-weighted images (DWI). Post-contrast T1-weighted sequences may be used selectively as an optional problem-solving tool rather than as a routine component of the protocol. The main aim of DWI is to act as an adjunct to determine tumor extent, helping discern tumor from fibrosis (especially in the post neoadjuvant treatment setting or after previous surgery). DWI can also facilitate lymph node detection. In selected cases, post-contrast series can assist in clarifying equivocal findings and indeterminate tissue planes—such as distinguishing subtle enhancing recurrence from scarring or evaluating suspected bladder or vaginal wall invasion—but they are not intended to replace DWI or contrast-enhanced CT for routine assessment of vascular involvement.

## Compartment-based surgical anatomy

**Figure 1:**
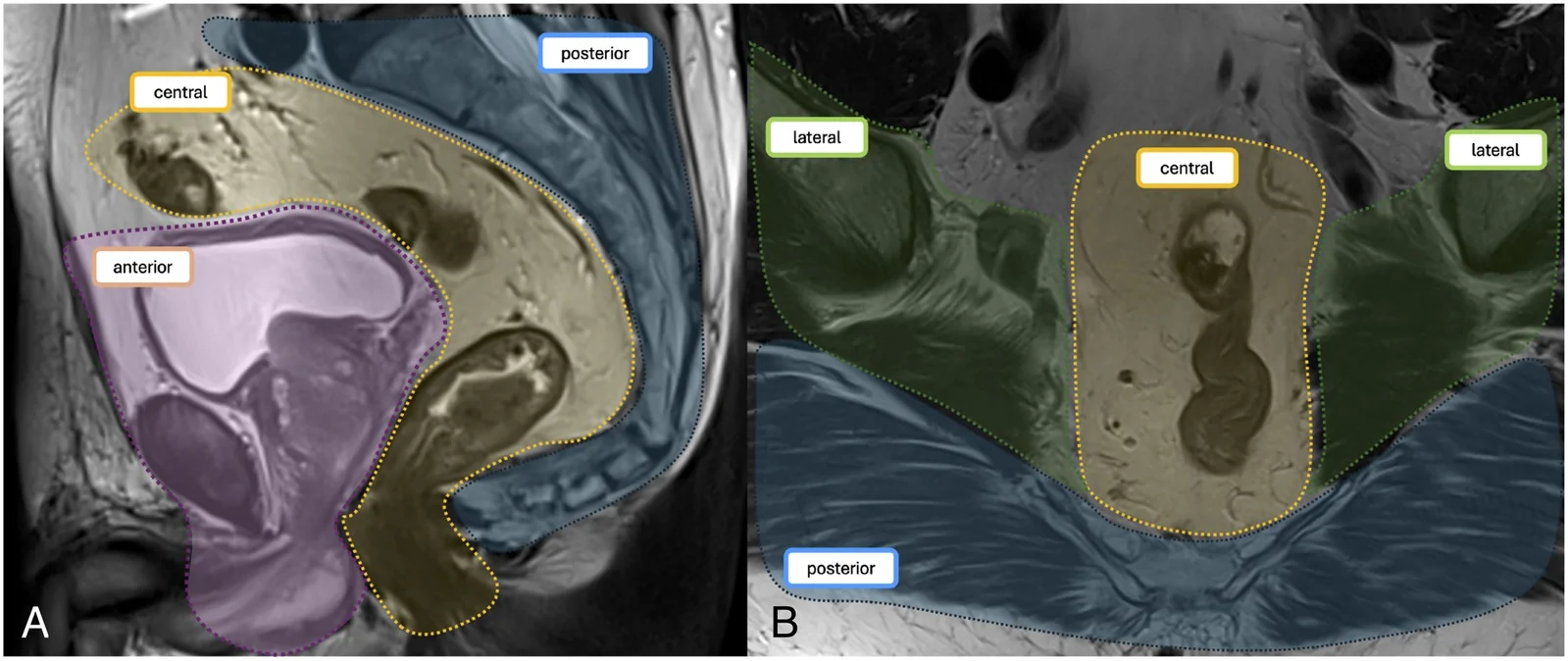
Compartment-based approach for describing pelvic patterns of disease on sagittal (A) and (B) axial plane. The central compartment (yellow), the anterior compartment (pink), the posterior compartment (blue) and the lateral compartment (green) are illustrated to guide consistent reporting of tumour location.

A compartment-based understanding of pelvic anatomy is essential for planning and performing PE. This section describes the anatomy and key structures of the central, anterior, posterior, and lateral compartments, highlighting their surgical relevance, implications for oncologic resection, including case examples and key pearls and pitfalls for radiological reporting to guide surgical planning (Figure 1).

### Central compartment

#### Contents

Rectum (including anastomosis, if present), mesorectum, the anal canal (including the internal and external anal sphincters), and levator ani muscles (Figure 1).

#### Anatomic overview

##### Rectum and mesorectum

The rectum is enclosed by the mesorectal fascia (MRF).^23–28^ Posterior to the MRF lies the parietal pelvic fascia over the presacral structures. The relatively avascular plane create a “holy plane” that facilitates clean dissection allowing total mesorectal excision (TME).^23–28^ Laterally, the MRF fuses with the pelvic sidewall fascia, while inferiorly it transitions into the levator ani and sphincter complex.^23–28^ The relationships of these planes are frequently distorted in recurrent disease introducing challenge when guiding assessment of resectability.

##### Anal canal and pelvic floor

The anal canal (approximately 2-4 cm) extends from the anorectal junction to the anal verge and is divided by the dentate line into upper and lower segments with distinct drainage pathways and implications for tumor spread.^29–34^ The smooth muscle internal sphincter represents continuation of the muscularis propria, compared to the striated external sphincter muscle which blends with the puborectalis, part of the levator ani, that forms a sling around the anorectal junction and is a key landmark for assessing anterior and posterior compartment involvement on MRI.^29–34^

#### Surgical considerations

The central compartment is the main site for primary and recurrent rectal cancers.^35–40^ The mesorectum and its fascia constitute the oncologic unit resected in TME, whereas primary tumors extending beyond this fascia or tumor recurrences often require wider “beyond TME resection.”^41^ Involvement of the anal sphincter or pelvic floor often mandates an abdominoperineal excision (APE) or an extra-levator excision (ELAPE).^42–44^ Prior surgery and radiotherapy make tumor delineation more challenging and increase the likelihood of requiring extended surgical margins. MRI signs of dense fibrosis or loss of fat planes can predict surgical difficulty, with increased bleeding risk requirement of more extensive resection.

#### Pearls and pitfalls for radiologic reporting

When reporting tumor distribution within the central compartment, 2 critical aspects must be evaluated systematically to guide surgical strategy and accurate staging:

##### Mesorectal fascia and beyond—implications for beyond TME surgery

In primary rectal cancer, when a tumor lies within 1 mm of the fascia, the risk of circumferential resection margin (CRM) involvement rises substantially and should be clearly reported.^45^ Direct invasion beyond the MRF into adjacent structures defines the T4b category and involvement of anterior compartment organs, the presacral fascia, pelvic sidewall, or neurovascular structures should be clearly highlighted to guide surgical planning. Any breach of the mesorectal envelope should be explicitly described, even in the absence of overt organ invasion, requiring a different surgical approach or team to offer an oncologic plane wider than standard TME.^46^

**Figure 2:**
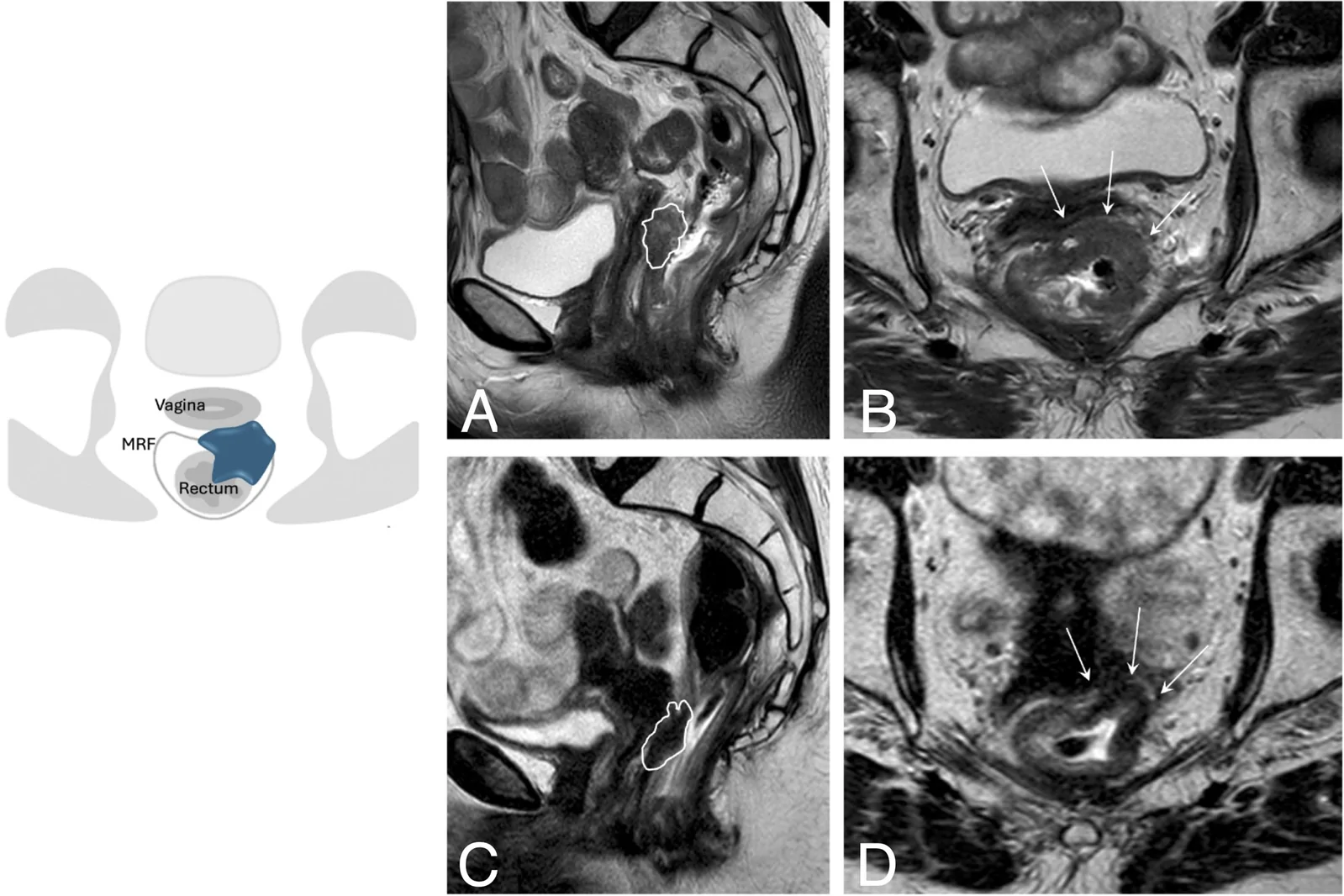
Female patient with an anastomotic recurrence following TME (low anterior resection). Baseline sagittal (A) and axial oblique (B) T2-weighted images show broad contact with the posterior vaginal wall (arrows). After chemoradiotherapy (C, D), fibrosis persists with no clear fat plane. Pathology confirmed vaginal wall invasion. **Key point**: loss of fat planes due to fibrosis after treatment does not indicate clear surgical margins.

In the setting of central recurrent disease occurring at the site of the anastomosis, T-categorization (as used for primary staging) is less applicable. Nevertheless, the radiology report must clearly state whether the recurrent tumor remains confined to the central compartment or extends into other compartments (Figure 2).^47^^,^^48^ After chemoradiation, distinguishing fibrosis from persistent tumor beyond the TME plane is a well-known challenge (Figure 2). Subtle, thin fibrotic strands—especially when no definite invasion was seen pretreatment—generally indicate a preserved margin, whereas bulky confluent fibrosis raises concern for persistent involvement and should prompt consideration of extended surgical planes.^47^^,^^48^

##### Anal canal involvement—implications for sphincter preservation

**Figure 3:**
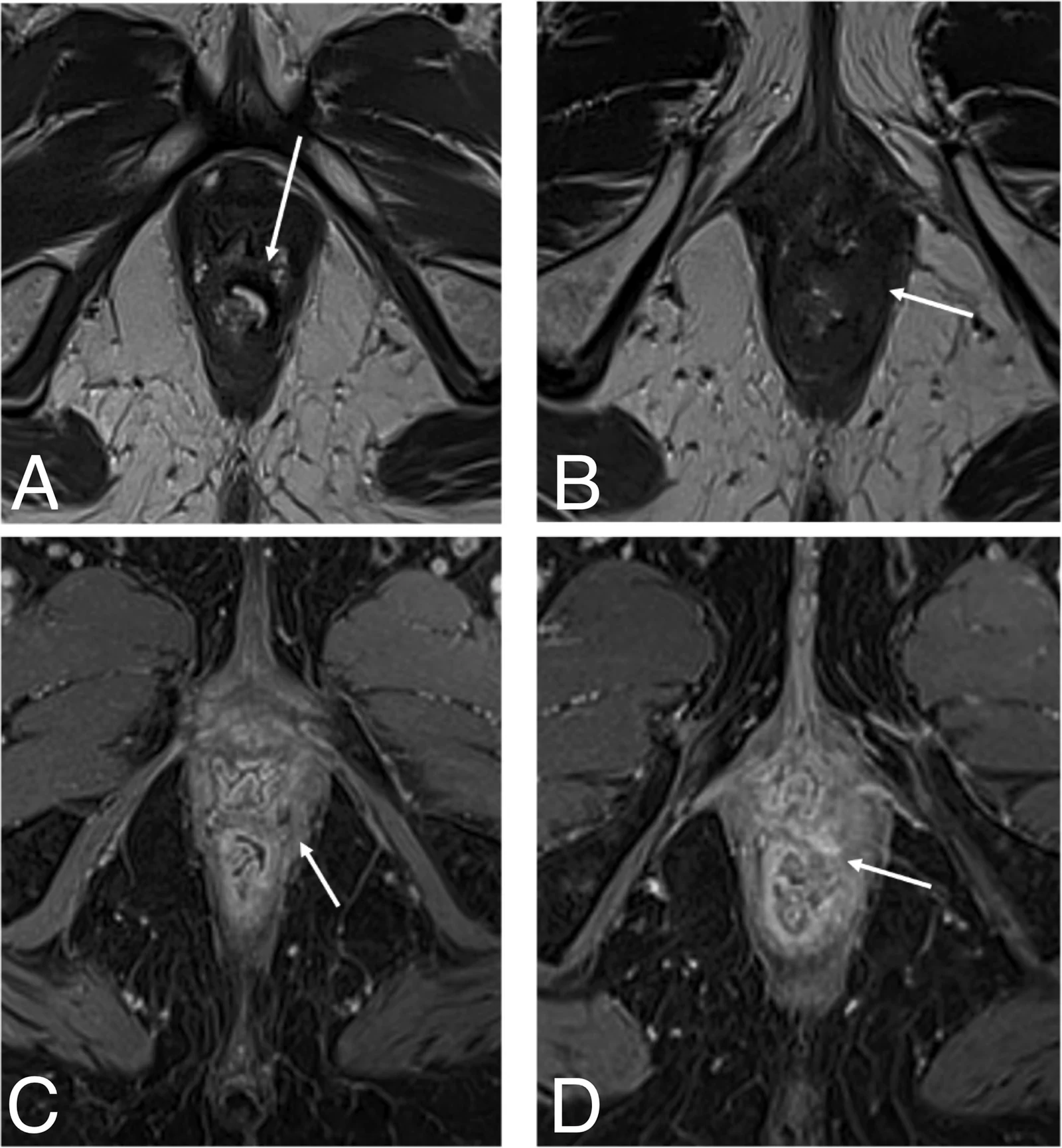
Female anal canal cancer patient initially managed with chemoradiation therapy. Follow up MRI at 9 months after chemoradiation therapy shows a complete response on axial oblique high resolution T2-weighted image (A, arrow) and post contrast axial oblique T1-weighted image (C, arrow). 18 months later, axial oblique high resolution T2-weighted image (B) and post contrast axial oblique T1-weighted image (D) demonstrate anterior local recurrence abutting the left puborectalis muscle and infiltrating the posterior vaginal wall (arrow). **Key point**: evaluation in true axial and axial oblique planes helps distinguish subtle regrowth from treated tissue, especially anteriorly.

Involvement of the anal canal is a key determinant of sphincter preservation. Radiologically, involvement of the internal sphincter (extension of the muscularis propria) differs from infiltration of the external sphincter or levator ani complex, which may necessitate more extensive resections such as an ELAPE.^49^^,^^50^ A common pitfall—particularly after neoadjuvant therapy—is underestimation of subtle involvement of the puborectalis sling or deep external sphincter fibers due to post-treatment edema or fibrosis. Careful evaluation on axial and especially coronal oblique T2-weighted images is essential, angled parallel to the axis of the anal canal (Figure 3).

#### Reporting essentials

- **Mesorectal fascia and Beyond:** Specify tumor contact or invasion of the MRF and adjacent structures.
- **Sphincter Complex:** Report involvement of internal/external sphincters, intersphincteric space or levator ani.

### Anterior compartment

#### Contents

Bladder, urethra, prostate, vas deferens and seminal vesicles (in men), uterus, vagina, ovaries, fallopian tubes (in women), pubic symphysis, superior and inferior pubic rami (Figure 1).

#### Anatomic overview

##### Bladder and urethra

In women the bladder dome, body, and trigone lie directly anterior to the cervix and upper vagina. The ureters pass just lateral to the cervix, crossing beneath the uterine artery (“water under the bridge”). The trigone is defined by the ureteric orifices and the internal urethral orifice.^51^^,^^52^ The female urethra (3-4 cm) runs within the anterior vaginal wall and is surrounded by the striated external sphincter, innervated by the pudendal nerve, while the male urethra has prostatic, membranous and penile components; the membranous segment is at risk of injury from its fixed position and proximity to the anal canal.

##### Uterus, vagina, and ovaries

The uterus is suspended by the broad ligament, which contains the fallopian tubes, ovarian ligament, round ligament, and associated vasculature—common pathways of parametrial spread.^51^^,^^53^^,^^54^ The rectovaginal septum and vesicovaginal space form fascial planes that guide dissection during anterior or central exenteration and are essential landmarks when assessing recurrence.^51^^,^^53^^,^^54^

##### Prostate and seminal vesicles

Denonviliers’ fascia separates the rectum from the prostate and seminal vesicles and may be infiltrated early by anterior rectal tumors. The seminal vesicles lie superior/posterior to the prostate and abut the bladder base and peritoneal reflection with close relationship to the ureters.^55^^,^^56^

##### Bones

The pubic symphysis and adjacent pubic rami form the anterior bony boundary. Their involvement, though uncommon, may necessitate partial pubectomy and influence reconstructive planning.

#### Surgical considerations

The corona mortis (“crown of death”) is a common variant of arterial and venous anatomy where there is an anastomosis between the external iliac or inferior epigastric vessels and the obturator vessels. These vessels are prone to injury with potential catastrophic bleeding as a consequence.^57^ Cross-sectional imaging can identify this arrangement preoperatively to anticipate planning and guide the dissection plane.^58^

Extent of anterior compartment involvement determines whether a partial or total PE is required.

- In men, the membranous urethra—short and fixed—is particularly vulnerable to injury during abdominoperineal resections.
- Trigone or urethral invasion generally mandates total cystectomy.
- Bladder dome involvement may allow partial cystectomy with preservation of urinary function.
- Isolated unilateral seminal vesicle involvement can sometimes be addressed with extended central resection without full exenteration.
- Distal ureteral involvement may be reconstructed using a Boari flap or psoas hitch.

Extended TME with limited (“shave”) posterior prostate resection has been described but is now discouraged due to high rates of positive margins and functional morbidity.^59^ As such, radical resection through total exenteration remains the standard of to ensure oncologic clearance.

#### Pearls and pitfalls for radiologic reporting

##### Bladder involvement

**Figure 4:**
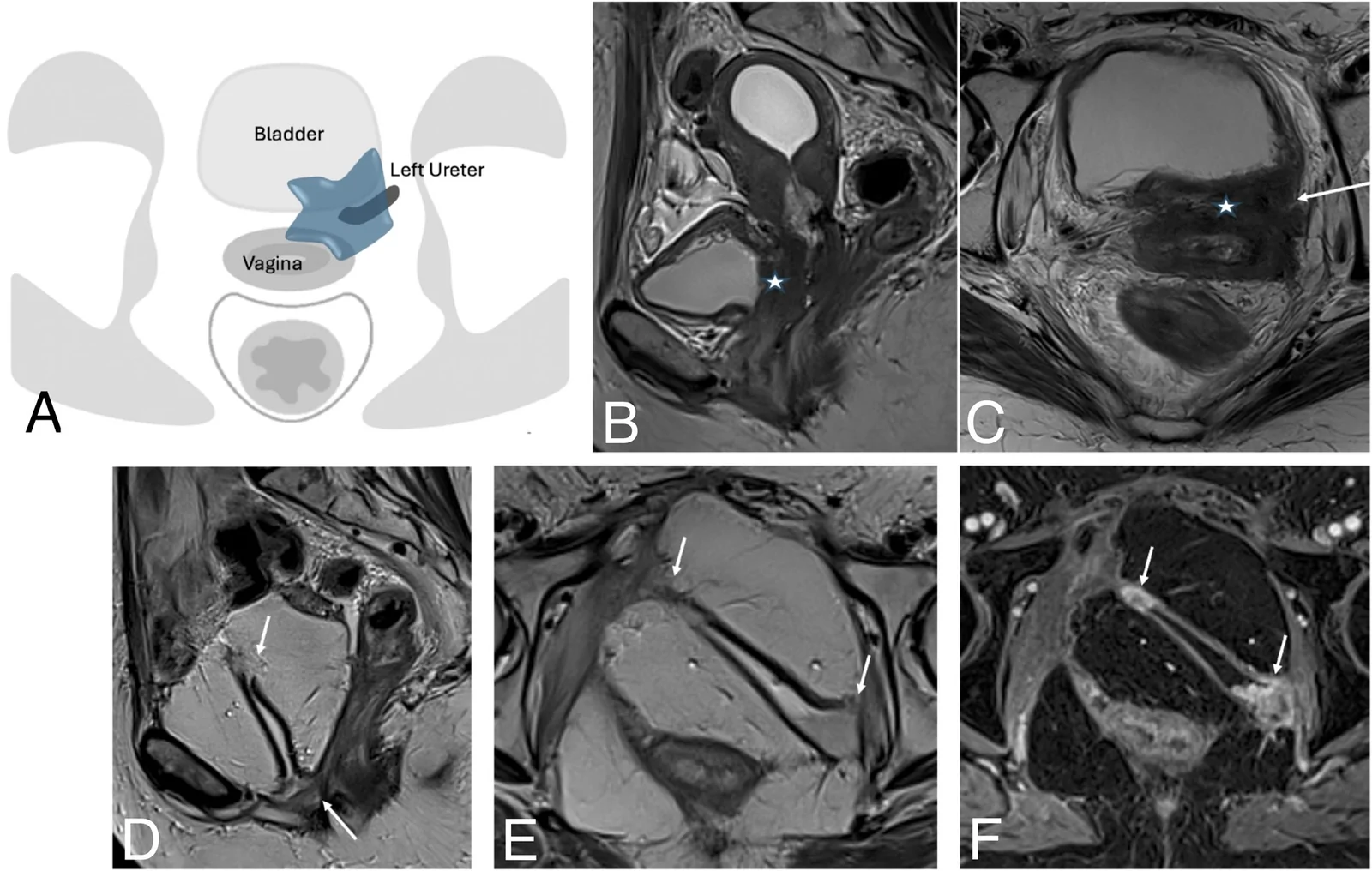
Female patient treated for a locally advanced cervical cancer (adenocarcinoma type) by chemoradiation therapy. Follow up at 18 months shows a large tumor recurrence extending within the bladder trigone, the triangular area of the bladder floor between the ureteric orifices and the internal urethral opening (B, sagittal and C, axial T2-weighted image, star). It is important to clearly describe involvement of the trigone as this indicates a need for complete cystectomy. The left ureter is also involved (schematic drawing [A], axial oblique T2-weighted image, [C] arrow). The patient underwent an anterior pelvic exenteration with a neovagina reconstruction and R0 resection. Two years later, sagittal (D) and axial (E) T2-weighted images demonstrate 2 areas of recurrence shown as hyperintense T2 signal (arrows) on the neovagina which demonstrate post contrast enhancement on T1 Fat Saturated sequence (F, arrows). Key point: clear identification of trigone involvement is crucial as it mandates cystectomy.

It is critical to clearly differentiate posterior bladder wall or dome invasion, from trigone involvement, as this will help the surgeon decide whether partial or total cystectomy is feasible (Figure 4). Furthermore, superficial invasion should be distinguished from macroscopic tumor extension into the muscular bladder wall or even bladder lumen. A classic pitfall overinterprets adhesions (or post-treatment) fibrosis as tumor extension. High-resolution T2 and contrast-enhanced imaging help confirm wall invasion.

##### Prostate and seminal vesicle involvement

**Figure 5:**
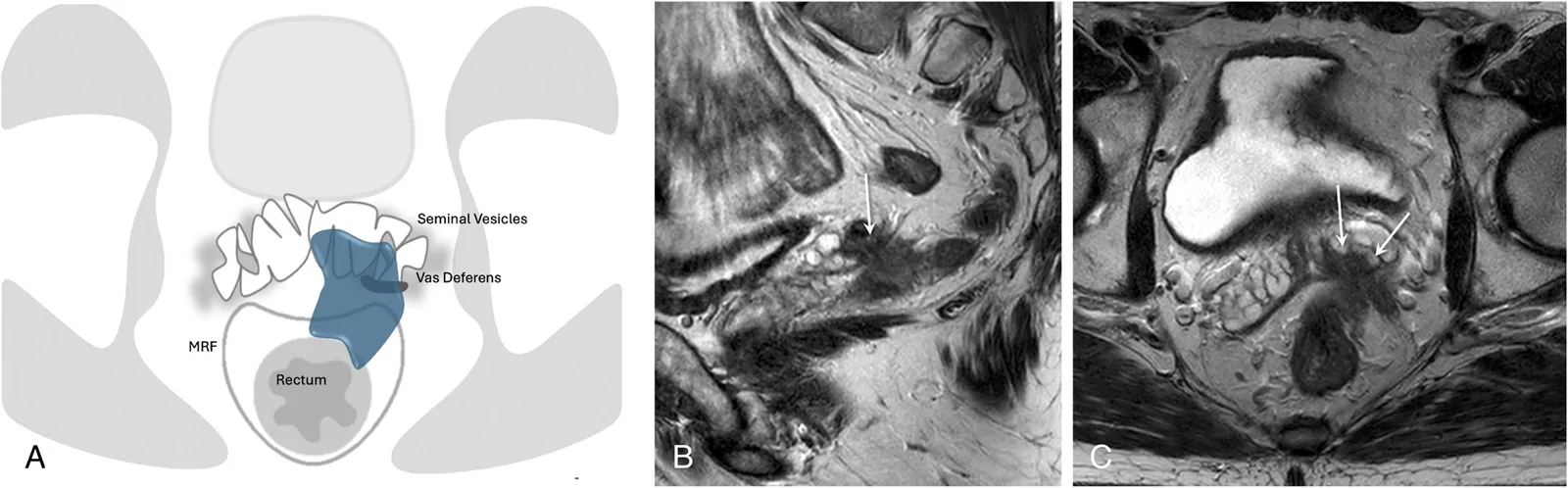
Schematic drawing (A) Sagittal (B) and axial (C) T2-weighted images of a male patient with a central rectal cancer recurrence following TME that extends into the anterior compartment with isolated involvement of the left seminal vesicle and vas deferens. Extended TME with left-sided seminal vesicle resection was performed. **Key point**: isolated seminal vesicle involvement may allow organ-preserving extended TME.

**Figure 6:**
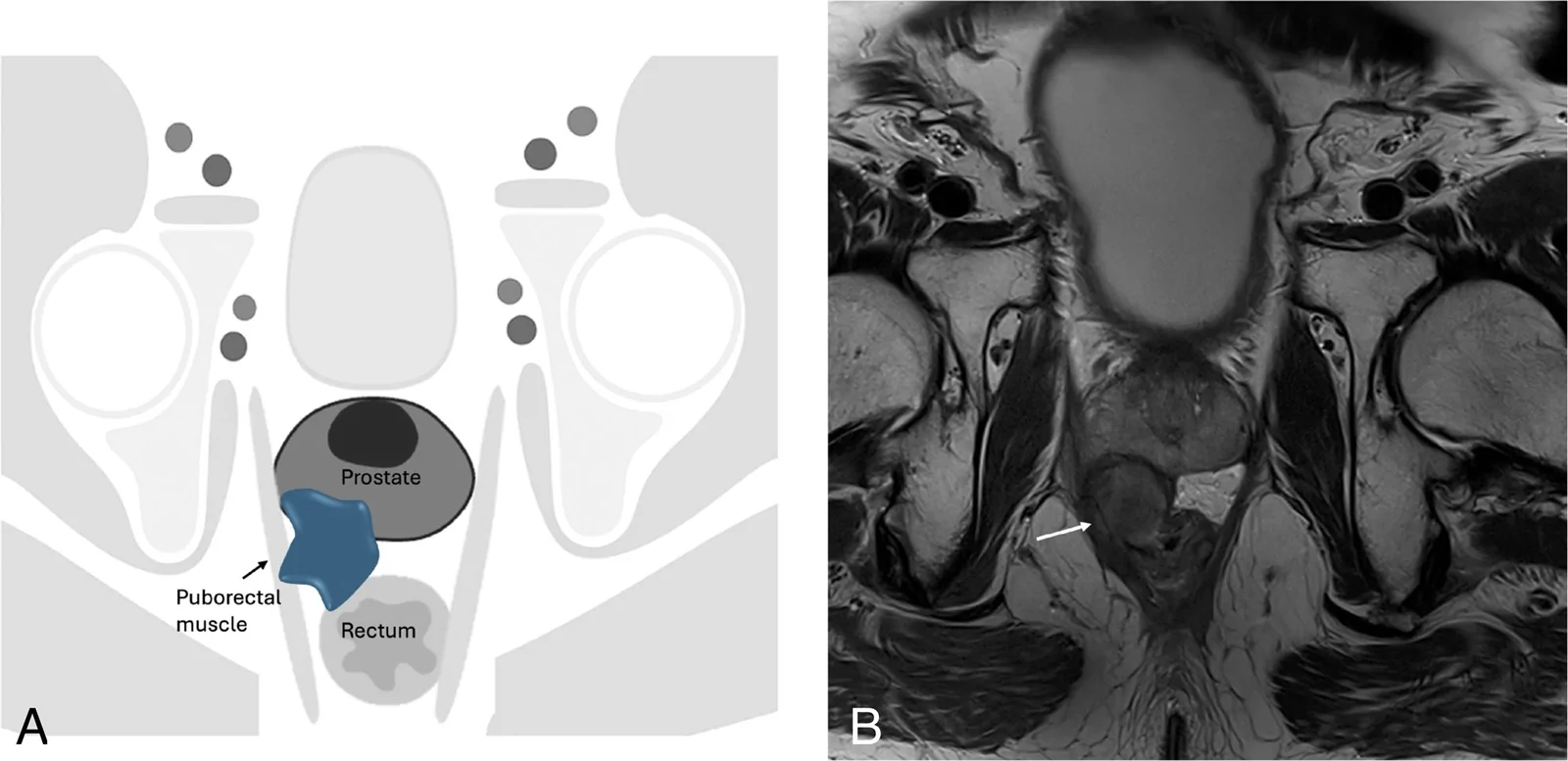
Schematic drawing (A) and Axial oblique (B) T2 weighted image of a male patient with a central rectal cancer recurrence following low TME extending into the anterior compartment, with involvement of the right puborectal muscle and prostate. Extended abdominoperineal resection with prostatectomy was performed. **Key point**: interruption of muscle architecture indicates infiltration and alters resectability.

In male patients, loss of the fat plane between tumor and prostate suggests possible invasion, but true prostatic infiltration (T4b) requires en bloc resection. Isolated seminal vesicle involvement may allow central resection without exenteration (Figures 5 and 6).

#### Reporting essentials

- Specify if posterior bladder wall, trigone, urethra, seminal vesicles (unilateral or bilateral), or prostate are involved,
- Avoid vague terms like “abutment”; prefer clear phrases (“loss of fat plane,” “definite invasion”),
- Use all planes systematically, particularly in post-treatment settings.

### Posterior compartment

#### Contents

Sacrum, coccyx, presacral fascia, sacrospinous and sacrotuberous ligaments, piriformis muscle, coccygeus, sciatic nerve and branches (Figure 1).

#### Anatomic overview

The posterior pelvic compartment consists of bony, ligamentous, muscular, and neurovascular structures critical for pelvic stability, autonomic function, and lower limb innervation. The sacrum (S1-S5) articulates with the iliac bones and transitions into the coccyx, which anchors pelvic floor musculature. The presacral fascia and associated ligaments form key planes for dissection and are frequently displaced or obliterated in recurrent disease (Table 1).

**Table 1:** Pelvic bony anatomy in PE: surgical and oncological significance.

Bone	Surgical/functional relevance	Interpretation pearls and implications for surgical planning
Sacrum	Level of sacral bone (and nerve) involvement determines **feasibility of nerve preservation** Level of sacral transection predicts **postoperative continence and ambulation**	**Cortical or** s**ubperiosteal involvement** → limited anterior sacral resection possible **Marrow invasion** → sacrectomy required **≥ S3 preserved** → continence and ambulation usually maintained **S1-S2 invasion** → major functional deficits; may preclude curative PE
Coccyx	Commonly resected in posterior PE to **facilitate surgical access to presacral space**; aids en bloc clearance in midline or presacral recurrences Resection associated with **minimal functional morbidity**	Involvement **rarely limits operability**
Pubic Symphysis	**Pelvic stability and reconstruction** planning depend on extent of involvement Key **landmark for anterior exenteration**	Involvement: Necessitates **partial pubectomy** Confirms **anterior compartment disease** Influences **urinary diversion planning and flap reconstruction**
Ischium	Defines **lateral compartment involvement** Impacts **feasibility of nerve and vessel preservation**	Involvement: Often requires **radical lateral wall resection** May necessitate **sacrifice of pudendal and/or obturator** structures
Iliac Bones (Ilium)	**Stability** of pelvic ring Involvement dictates **need for skeletal reconstruction** (and vascular ligation/reconstruction in case of encasement)	Extent of involvement determines **feasibility of en bloc resection** High iliac involvement → bone resection often feasible
Pararectal and Paravesical Spaces:	Critical areas to determine tumor boundaries and **need for lymph node dissection**	Involvement: Indicates lateral compartment involvement May necessitate lateral lymph node dissection
Obturator Space	Defines zone for **lateral sidewall dissection**	Involvement: Necessitates **extended lateral dissection** Induces risk of **obturator nerve sacrifice**

##### Fascia, ligaments, and muscles

**Figure 7:**
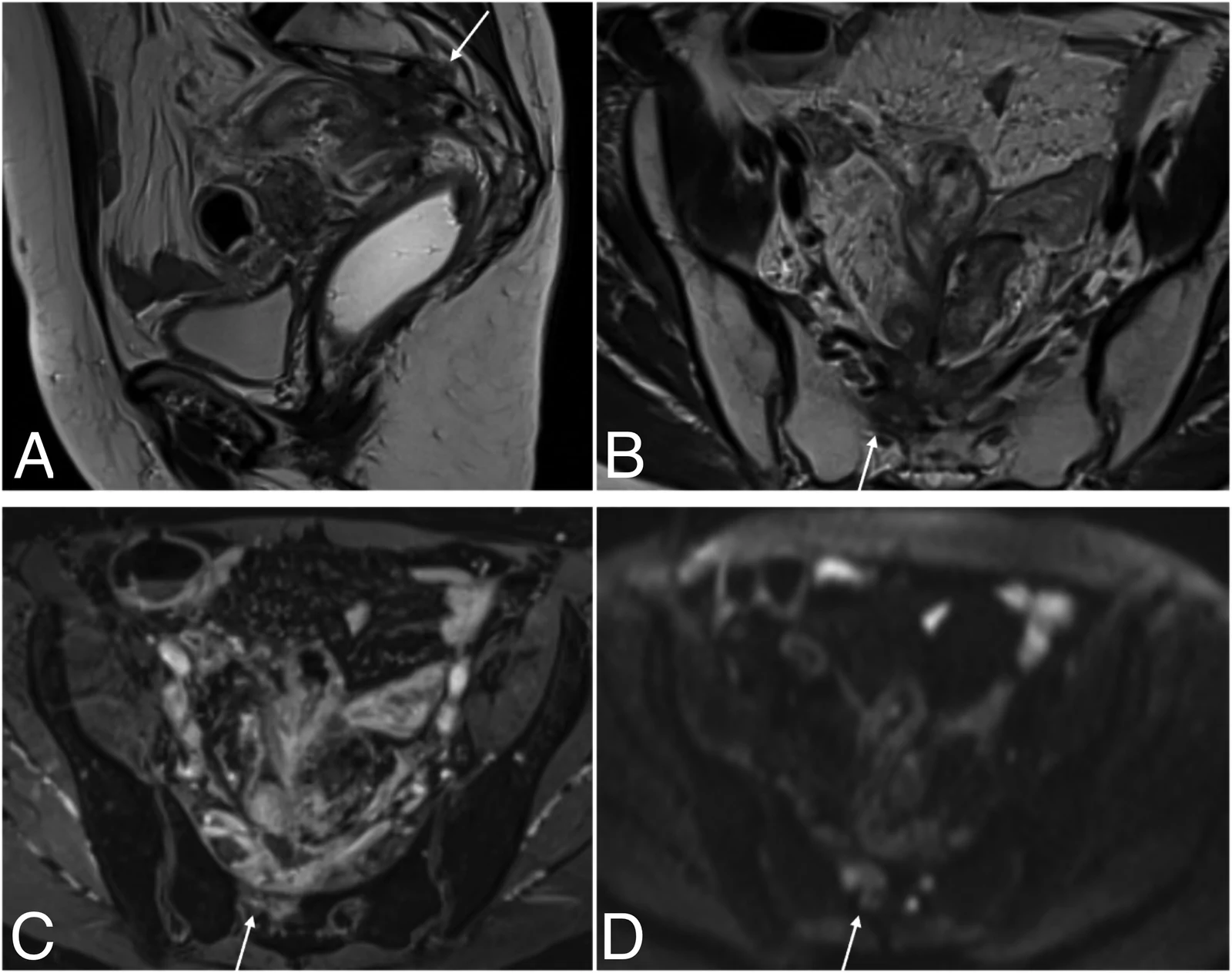
Recurrent rectal cancer with osseous extension into S2-S3 vertebrae on sagittal (A) and axial (B) T2-weighted images, confirmed by post-contrast T1 (C) and DWI (D). **Key point**: high sacral involvement (S2-S3) precludes en bloc exenteration.

**Figure 8:**
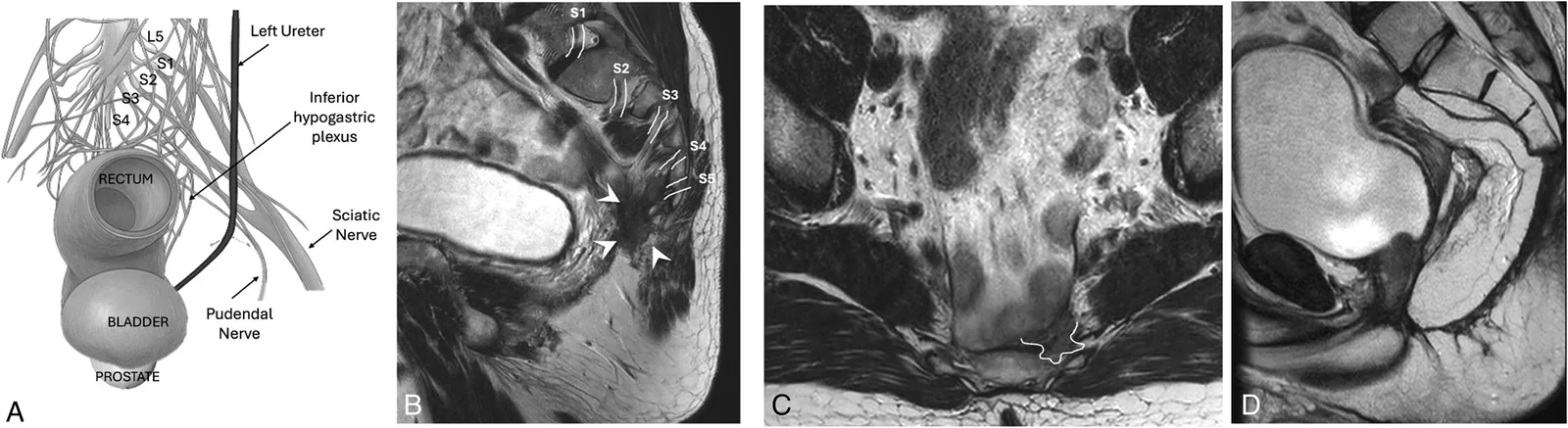
Schematic drawing showing the nerve roots of the pelvis (A). Sagittal (B) and axial (C) T2-weighted MR images of a male patient showing a recurrent rectal cancer in the posterior compartment following abdominoperineal resection. The nerve roots of S1-S3 are not involved, but there is invasion of the S4-S5 roots on the left side, extending into the S4 neuroforamen. Exenteration with sacrectomy including S4-S5 and part of S3 was performed; D shows the postoperative situation with an Oblique Rectus Abdominis Myocutaneous (ORAM) flap reconstruction in place to fill the pelvic defect after surgery. **Key point**: lower sacral involvement (S4-S5) may still be operable with tailored sacrectomy.

Sacral nerve roots (Figures 7 and 8): The sacral roots (S1-S5) exit through the anterior sacral foramina and contribute to major pelvic nerves (Tables 2 and 3).^60^

**Table 2:** Pelvic fascial anatomy: surgical and oncological significance.

Fascial structure/space	Surgical/functional relevance	Interpretation pearls and implications for surgical planning
Mesorectal Fascia (Visceral Mesorectal Fascia)	Defines **TME plane** and CRM	Tumor-MRF distance <1 mm = **involved MRF** → need for neoadjuvant treatment and/or extended resection **Invasion beyond MRF** → beyond‑TME resection.
Parietal Pelvic Fascia	Establishes **lateral limit** of standard pelvic dissection Reference for **sidewall disease**	Thickening or loss of plane indicates **sidewall extension** May necessitate extended **lateral wall resection**
Presacral Fascia	Critical dissection plane in **posterior pelvic surgery** and high subcortical sacral (HiSS) resections Major **bleeding risk**	Involvement: Implies **posterior compartment spread** May necessitate **sacral resection**
Rectoprostatic (Denonvilliers’) Fascia	Determines feasibility of organ‑preserving vs en bloc **urogenital resection**	Loss of plane suggests **urogenital invasion** often mandates **anterior pelvic exenteration**
Rectovaginal and Vesicovaginal Septa	Natural surgical corridors	Loss of planes suggests **anterior organ invasion** Often mandates en bloc resection
Retropubic Space (Space of Retzius)	Allows **bladder mobilization** Critical area for **vascular awareness** during anterior approaches	When tumor involvement **extends to pubic symphysis**, **partial pubectomy** may be required
Pararectal and Paravesical Spaces:	Critical areas to determine tumor boundaries and **need for lymph node dissection**	Involvement: Indicates lateral compartment involvement May necessitate **lateral lymph node dissection**
Obturator Space	Defines zone for **lateral sidewall dissection**	Involvement: Necessitates **extended lateral dissection** Induces risk of **obturator nerve sacrifice**

**Table 3:** Pelvic neural anatomy: surgical and oncological significance.

Nerve	Surgical/functional relevance	Interpretation pearls and implications for surgical planning
Obturator nerve (somatic)	Critical structure in **lateral lymph node dissection and sidewall surgery** Injury results in **loss of thigh adduction and gait instability**	Involvement usually mandates nerve sacrifice
Sciatic nerve (somatic)	Critical determinant of **limb function**	Involvement: Defines feasibility of posterior/lateral resections Generally **contraindicates curative surgery** due to major functional morbidity
Pudendal nerve (somatic)	Critical for **continence** Key landmark in **sphincter-preserving** surgery	Involvement: Drives choice between **sphincter preservation vs ELAPE** Often necessitates **permanent stoma**
Hypogastric nerves (sympathetic)	Often sacrificed in posterior compartment resections Injury results in **mild functional morbidity** incl. mild sexual dysfunction (erectile function often preserved) and mild urinary dysfunction	Involvement indicates deeper posterior disease Sacrifice (with **mild functional morbidity**) often considered acceptable to secure R0 margins
Pelvic splanchnic nerves (parasympathetic)	At risk during anterior/lateral dissection Injury results in **urinary and bowel dysfunction**	Tumor **extension into pararectal/paravesical spaces** suggests involvement → often requires sacrifice Explicit **preoperative functional counselling** required
Inferior hypogastric plexus (mixed)	Defines functional **boundary of lateral and anterior compartments** Injury results in **major functional morbidity** incl. severe bladder, bowel and sexual dysfunction	Involvement: Defines **high‑risk lateral disease** Typically requires en bloc resection with **predictable autonomic deficit** Explicit **preoperative functional counselling** required

- **Sciatic nerve (L4-S3):** exits the pelvis below the piriformis and is critical for lower limb function.
- **Pudendal nerve (S2-S4):** responsible for continence, running through the sciatic foramina and Alcock’s canal.

The degree of nerve sacrifice is balanced with acceptable postoperative function and morbidity.

#### Surgical considerations

Posterior compartment involvement is most often seen in recurrent rectal cancer or primary tumors extending beyond the posterior MRF.^61^^,^^62^

Involvement of the sacrum, coccyx, or pelvic ligaments may require posterior pelvic wall resection, including coccygectomy or sacrectomy—operations associated with substantial morbidity, particularly regarding neurological impairment and wound healing.

When disease is confined to the presacral fascia without bone invasion, limited subperiosteal resection, including high subcortical sacral resection (HiSS) may be possible. Once the tumor breaches the periosteum and involves sacral marrow, true sacrectomy becomes necessary.

- Resections at or below S3 generally preserve ambulation and continence.
- Resections involving S1-S2 carry a high risk of bladder, bowel, and lower-limb dysfunction and may render exenteration non-viable.

Hemi-sacrectomy or angled osteotomy may allow preservation of contralateral sacral roots in selected cases. Accurate MRI delineation of cortical vs marrow invasion is therefore crucial to determining whether a function-preserving posterior resection is achievable. Ultimately, decisions must balance the goal of achieving an R0 resection against the functional cost of high sacral division.^61-64^

#### Pearls and pitfalls for radiologic reporting

##### Sacral and coccygeal involvement

**Figure 9:**
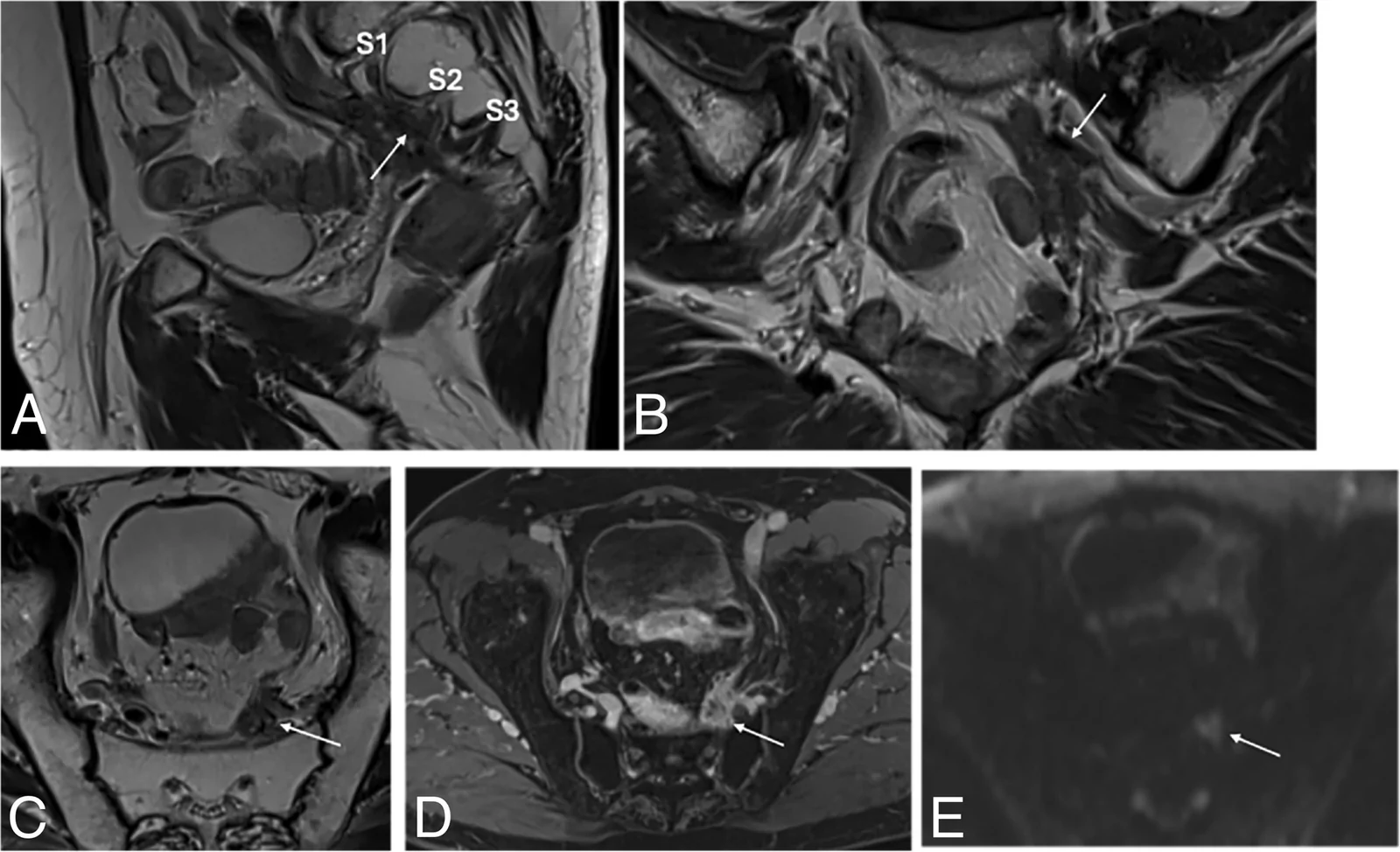
Sagittal (A), coronal (B) and axial (C) T2-weighted MR images of a female patient showing a recurrent rectal cancer in the posterior compartment following abdominoperineal resection. Note that the recurrence is difficult to depict on T2-weighted images and is better visualized on the post-contrast T1-weighted image (D) and DWI (E). The left S1-S2 nerve roots are involved. **Key point**: recurrence near upper sacral levels (S1–S2) is typically not resectable.

It is important to differentiate superficial bone contact from true cortical or marrow invasion. Inflammatory changes or fibrosis may be misinterpreted as bone invasion. Combined use of sagittal and axial T2 (in plane with the intervertebral discs), fat-saturated, and post-contrast sequences may aid in evaluating marrow signal changes and cortical disruption. In addition, CT images (when available) can confirm suspected cortical disruption.^14^ The level of sacral involvement (S1/2 vs S3 or below) dictates postoperative function as well as surgical approach (above S3 may require prone sacrectomy) and should be clearly specified in the report (Figures 7-9).^65^

##### Sacral nerve root and plexus involvement

Sacral nerve roots (S1-S5) and pudendal nerve (Figures 7-9) must be assessed for invasion or encasement, as their involvement affects continence, sexual function, and lower limb motor control. On MRI, nerve invasion appears as loss of fat planes, abnormal thickening, or discontinuity, best seen on high-resolution T2W-images. When reporting neural involvement, radiologists should specify whether invasion extends into the neural foramina or involves nerve roots, the distal course of the sacral nerves or the sacral plexus (Figures 7-9).^66^

#### Reporting essentials

- Differentiate presacral fascia involvement vs bony sacral invasion
- Specify the exact sacral levels affected
- Clearly describe nerve root or plexus invasion
- Consider 3D planning (MRI/CT) when sacrectomy or complex resections are anticipated.

### Lateral compartment

#### Contents

Ureters, internal iliac arteries and veins and their branches, obturator nerve and muscles, iliac bones, ischial spine, ischium, sacral nerve roots (Figure 1).

#### Anatomic overview

##### Nerves and muscles

The obturator nerve (L2-L4) follows the lateral pelvic wall and exits via the obturator canal with the obturator vessels (Table 3). The pudendal nerve (S2-4) courses along the sacrospinous ligament and reenters the pelvis through the lesser sciatic foramen before entering Alcock’s canal to supply the perineum and external sphincters. The obturator internus muscle forms much of the lateral pelvic wall with origin from the ischium and contact with the ischial spine.^63^^,^^64^^,^^67^

##### Vessels

The internal iliac artery divides into anterior and posterior trunks, giving rise to vesical, uterine/prostatic, middle rectal, and superior and inferior gluteal branches (Table 4). Its venous counterpart forms a complex plexus with highly variable tributaries—one of the major bleeding challenges of lateral resections.

**Table 4:** Pelvic vascular anatomy: surgical and oncological significance.

Vessel	Surgical/functional relevance	Interpretation pearls and implications for surgical planning
External Iliac Artery & Vein	Principal inflow/outflow to lower limb Critical **landmark for lateral compartment dissection**	Resection feasible with reconstruction **Venous resection is more technically challenging** than arterial → often defines non‑preservable lateral compartment disease.
Internal Iliac Artery & Vein	Supply pelvic organs, perineum and gluteal region Ligation significantly **alters pelvic perfusion**	**Unilateral** ligation usually tolerated **Bilateral** ligation risks gluteal necrosis unless posterior division preserved Venous involvement indicates major **bleeding risk**
Obturator Artery & Vein	Key vascular landmark in lateral lymphadenectomy Presence of corona mortis increases intraoperative bleeding risk	Often sacrificed during lymphadenectomy Reporting of **corona mortis** is critical to flag increased bleeding risk.
Superior Gluteal Artery	Critical for **gluteal muscle and flap perfusion** Critical vessel in posterior compartment surgery (high bleeding risk)	Involvement suggests **posterior compartment invasion** Injury risks **massive hemorrhage** and **gluteal flap failure**
Inferior Gluteal Artery	Landmark for defining sciatic notch anatomy during posterior approaches Sciatic nerve devascularization results in **major functional morbidity** incl. loss of motor function (gait impairment), loss of sensory function and reflexes	Encasement common in sciatic notch recurrences Resection risks **sciatic nerve devascularization** and **major bleeding**
Middle Sacral Artery	Guides midline presacral dissection Frequent source of bleeding in sacral surgery	Commonly ligated during sacrectomy Injury associated with **massive presacral hemorrhage**

##### Ureters

The ureter courses medially across the pelvic brim, enveloped by parietal pelvic fascia, and lies close to the ovarian and uterine vessels in women.

##### Lateral lymph node compartments

Patterns of nodal spread differ by tumor type, but in rectal cancer the obturator and internal iliac nodes are considered locoregional.^68^ Their involvement has major implications for treatment, often prompting TME with lateral lymph node dissection. The anatomical limits of these nodal basins have been rigorously defined by Ogura et al.^35^^,^^69^

#### Surgical considerations

The lateral compartment is the among the most technically demanding areas of pelvic surgery due to densely packed vessels, nerves, bony structures, and lymphatic stations. Multi-visceral resections with en bloc resection of the lateral compartment or lateral lymphadenectomy require meticulous knowledge of individual variants to preserve function and achieve negative margins.

- Vessels:

Arterial encasement is typically more manageable than venous due to the complex, low-pressure internal iliac venous plexus.

- **Internal iliac artery ligation** (unilateral or bilateral) is generally well tolerated; preserving the posterior trunk helps prevent buttock claudication, especially when gluteal flaps are planned.
- **External iliac vessel involvement** may require segmental resection and reconstruction, depending on local expertise.
- **Venous reconstruction** is controversial; collateral pathways are often sufficient when epigastric veins are preserved.

Disease involving the common iliac system may require bespoke reconstructive strategies.^70^^,^^71^

- Lymph nodes:

In cases of malignant lateral nodes, formal compartmental dissection is preferred. Historical “node picking” is no longer recommended due to high recurrence rates.^72^

- Ureter involvement:

When ureteral involvement occurs, the level relative to the iliac bifurcation determines the ability to reconstruct:

- Below the bifurcation → often feasible for reimplantation.
- Above the bifurcation → may require nephrectomy or urinary diversion (eg, ileal conduit).

Extended lateral sidewall excision may be required for lateral invasion (seen in about 20%-30% of recurrent rectal cancers). Resection may involve the internal iliac vessels, obturator structures, or iliac bone. Preoperative embolization or stenting can reduce intraoperative bleeding risk.

#### Pearls and pitfalls for radiologic reporting

##### Vessel involvement

**Figure 10:**
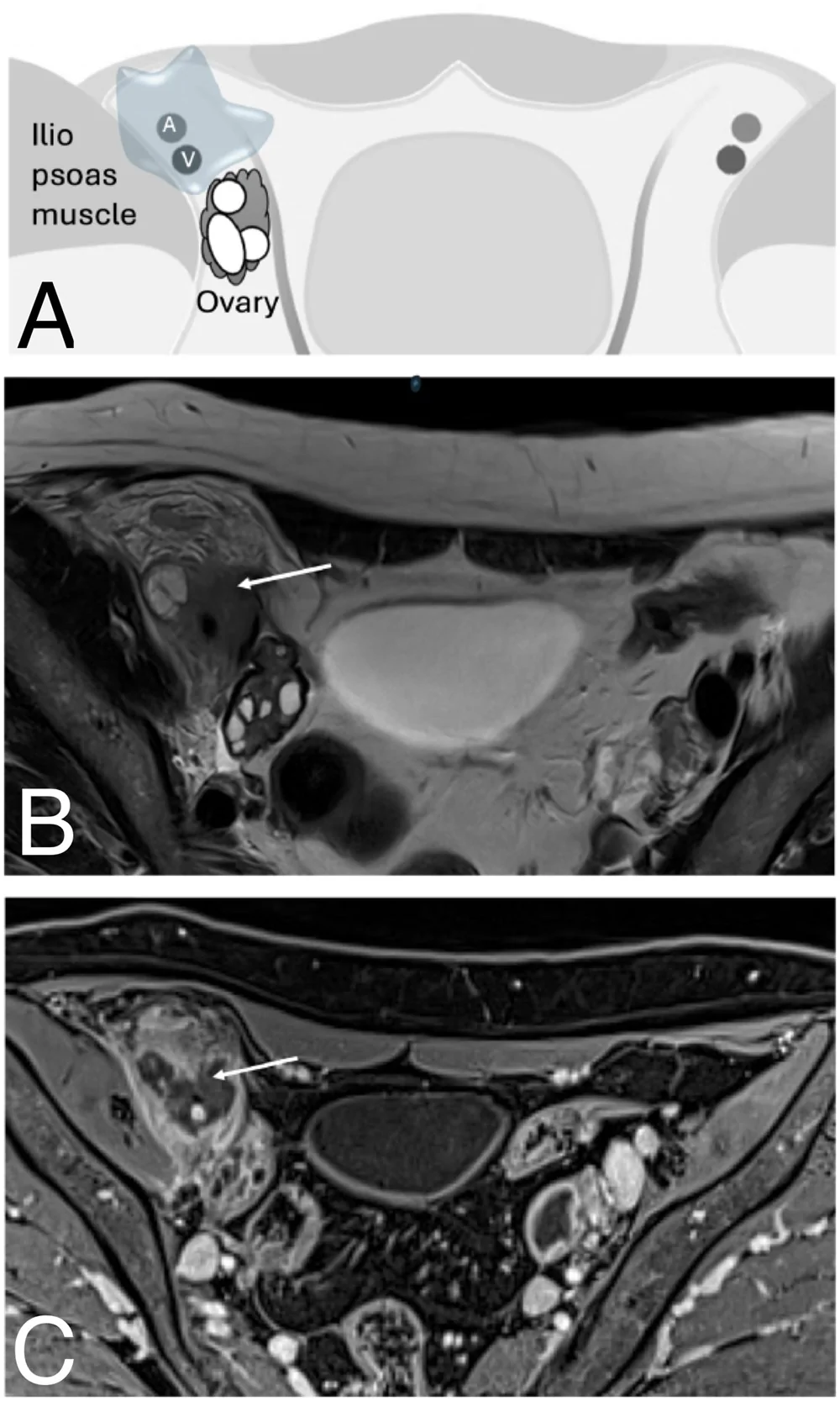
Schematic drawing (A), axial oblique (B) T2 and post contrast T1-weighted (C) images of a female patient with a right lateropelvic recurrence (nodal) of a cervical cancer centered between the right EIA and vein. Note the thin hyperintense T2 fat plane between the tumor recurrence and the right ovary ruling out infiltration at this level. **Key point**: preserved fat planes help exclude ovarian infiltration.

Reports should clearly specify whether the internal iliac artery, vein, or their branches are affected, and indicate whether disease lies within the anterior or posterior trunk (eg, superior/inferior gluteal, middle rectal, uterine/prostatic branches) (Figure 10).^14^^,^^17^^,^^73^ The degree of arterial or venous encasement should be described (eg, >180° contact), along with any signs of luminal narrowing, occlusion, or displacement. Because venous anatomy is highly variable and the venous plexus is prone to subtle invasion, radiologists should comment on indirect markers of venous compromise such as early venous filling, collateral pathways, or asymmetric congestion. Standard MRI may underestimate small-volume venous involvement; in selected cases, contrast-enhanced CT can provide better delineation of venous anatomy.

##### Lymph nodes

Lymph node assessment should specify involvement within the obturator, internal, external, presacral, and common iliac regions, detailing size, number, and proximity to vessels or nerves. Because metastases may extend above L5/S1, imaging should cover the common iliac and para-aortic areas when indicated.

##### Nerve and bone involvement

**Figure 11:**
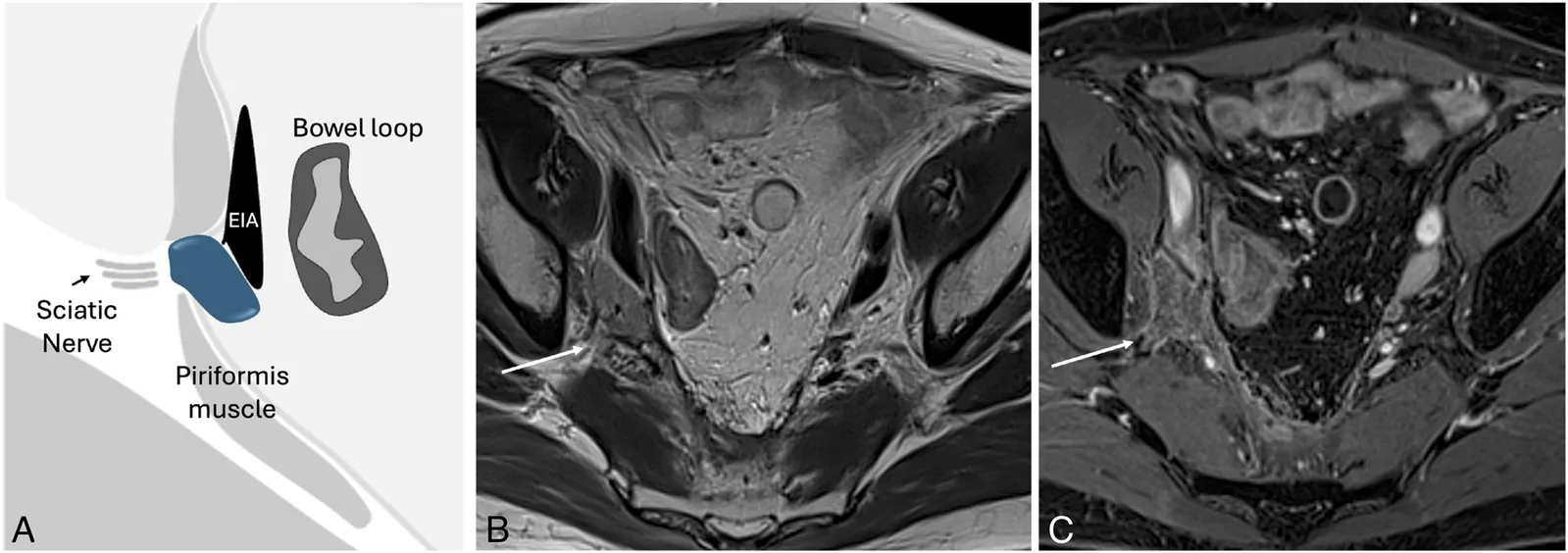
Schematic drawing (A), axial oblique (B) T2 and post contrast T1(C)-weighted images of a female patient with a right lateropelvic recurrence of a rectal cancer (who underwent a previous extralevator abdominoperineal resection). The recurrence is centered on the right sciatic nerve which makes resection not feasible as this would cause sacrifice of the sciatic nerve and result in major functional impairment of the lower limb. Note the thin hyperintense T2 fat plane between the tumor recurrence and the right EIA ruling out infiltration at this level. **Key point**: sciatic nerve involvement generally contraindicates radical resection.

**Figure 12:**
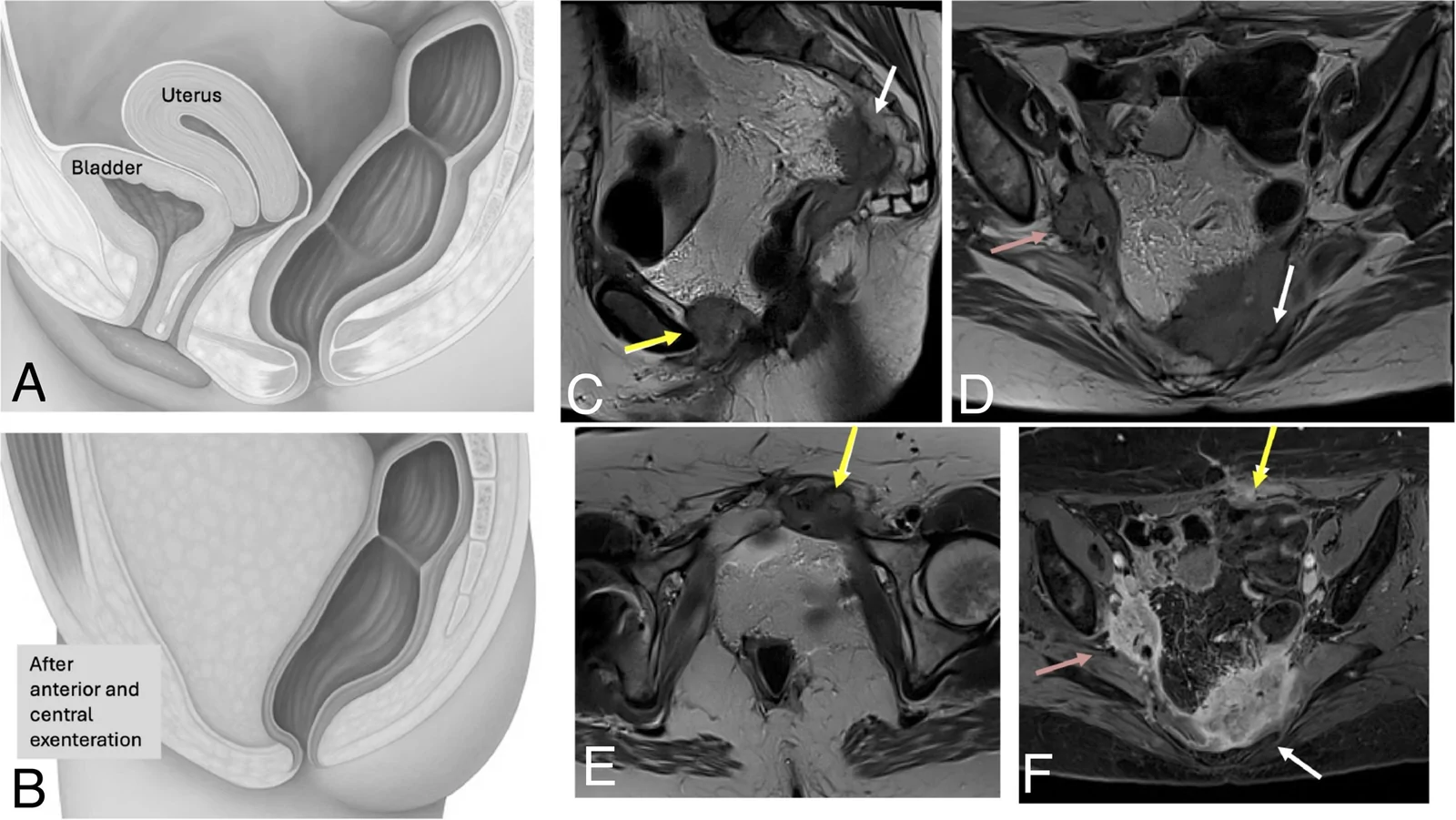
Schematic drawings of sagittal pelvis before (A) and after anterior exenteration (B). Sagittal (C) and axial (D, E) T2- and contrast-enhanced T1-weighted (F) MR images of a female patient with recurrent cervical cancer who had previously undergone anterior and central pelvic exenteration. The images demonstrate multifocal recurrence involving the left pubic symphysis (yellow arrow), the sacrum (white arrow), and the right lateral pelvic space with invasion of the obturator vessels and ischiatic fossa (pink arrow). **Key point**: multifocal recurrence across compartments often limits resectability despite prior exenteration.

Assessment of the obturator nerve, sciatic nerve, sacral plexus, and hypogastric nerves is essential, as invasion of these structures often dictates whether curative resection is feasible (Figures 11 and 12). Perineural involvement may be extremely subtle; careful evaluation of fascicular asymmetry, obliteration of fat planes, and focal enlargement of neural pathways on standard high-resolution sequences is therefore critical.

For osseous structures, radiologists should evaluate both cortical integrity and early marrow signal changes. Low T1 signal combined with high signal on fat-suppressed T2-weighted sequences may represent early bone involvement—even before cortical breach—and should be highlighted, as these findings influence decisions regarding sacrectomy or sidewall resection.

#### Reporting essentials

- Vessels: clearly specify iliac artery/vein involvement, including identification of anterior or posterior trunk branches affected (eg, superior/inferior gluteal, middle rectal, uterine/prostatic arteries) and extent of encasement (>180° contact) or obliteration.
- Lymph nodes: clearly report nodal involvement in the obturator, internal iliac, external iliac, presacral, and common iliac regions, including the size, number, and location of suspicious nodes, and their proximity to major vessels and nerves.
- Nerves: carefully assess and report tumor proximity or invasion of the obturator nerve, hypogastric nerve, sciatic nerve or sacral plexus.
- Bones: assess and report involvement of the ischial spine, sacrospinous ligament, or iliac bone, guiding the need for bone excision.

## Anatomic considerations for plastic reconstructive surgery

PE creates complex pelvic, perineal, and bony defects that often require flap-based reconstruction. While a detailed surgical review is beyond the scope of this paper, radiologists should be familiar with the most common reconstructive options to support preoperative planning and postoperative assessment.^74^

ObliqueRectus Abdominis Myocutaneous (ORAM) and Vertical Rectus Abdominis Myocutaneous (VRAM) flaps are frequently used for large perineal defects, providing well-vascularized tissue to obliterate dead space and reconstruct structures such as the vagina (Figure 4).^75^^,^^76^ They are supplied by the inferior epigastric vessels, which may be compromised by prior surgery, stomas, or radiotherapy. Preoperative imaging should assess vascular patency and local tissue quality. On MRI, the muscular component shows low T2 signal early, with progressive fatty replacement over time.^25^^,^^77^

Omentoplasty uses the highly vascularized greater omentum to fill smaller defects or cover irradiated or infected areas. It appears predominantly fatty on MRI. Imaging can detect complications such as insufficient volume, infection, or rare ischemia.^78^

Thigh flaps, typically based on branches of the profunda femoris artery, provide reliable soft-tissue bulk while preserving the abdominal wall and are particularly useful in patients with multiple stomas. Bilateral flaps can increase volume.

Gluteal flaps offer robust cutaneous coverage and can be used for vaginal reconstruction but depend on intact gluteal vascular supply and require prone positioning.

## Imaging after neoadjuvant therapy

Patients undergoing PE often receive neoadjuvant therapy (typically a combination of chemotherapy and radiotherapy) with the goal of downsizing the tumor and improving the likelihood of achieving an R0 resection. While effective in reducing tumor burden, these treatments frequently induce fibrotic changes that complicate subsequent imaging interpretation. In primary tumor post-treatment staging, numerous studies have demonstrated that conventional anatomic MRI struggles to differentiate viable tumor from fibrosis. The addition of diffusion-weighted imaging (DWI) has been shown to improve accuracy by helping to identify residual tumor and distinguish complete from incomplete responders.^79–82^

In the context of recurrent disease, imaging after neoadjuvant treatment is even more challenging.^83^ Recurrent tumors are often less well-defined, with more aggressive and infiltrative growth patterns, and these features are further obscured by treatment-induced fibrosis.^73^ Current response-grading tools, such as the MRI tumor regression grade (mrTRG), have shown limited utility in this setting.^79^ The primary focus of post-treatment imaging is accurate surgical planning rather than assessment of complete response, as the window during which these patients remain candidates for surgery is often limited.

Given these limitations, radiologists should aim to provide a detailed assessment of fibrotic changes in their restaging reports. It is essential to describe the extent and distribution of fibrosis, including which organs or compartments are involved, while clearly acknowledging the possibility of persistent tumor within these areas. This nuanced reporting is crucial to guide surgical planning and optimize oncologic outcomes.

### Reporting essentials after neoadjuvant treatment

- Combine anatomic (T2-weighted) assessment with DWI to enable better differentiation between residual tumor and fibrosis
- Include a clear description of the extent of tumoral as well as fibrotic involvement in the report as both will impact surgical planning

## Conclusion

Reporting essentials using compartment-based approach–detailing the involvement of specific anatomical organs and structure within each compartment is summarized in Table 5. A thorough understanding of pelvic anatomy is essential for successful PE. Knowledge of the spatial relationships between bones, vessels, nerves, and fascial structures facilitates complete tumor resection while minimizing morbidity when function can be preserved. As surgical and imaging techniques evolve, detailed preoperative imaging, multidisciplinary planning, and anatomic precision remain central to safe and effective procedures. The growing use of augmented reality in robotic surgery—integrating radiologic images directly into the operative field—further highlights the critical role of advanced imaging and meticulous radiologic assessment in guiding these complex interventions.

**Table 5:** Key items from ESGAR-SAR-ESUR-PelvEx structured reporting template for pelvic exenteration.^17^

I—Tumor description (for multifocal disease, report each lesion separately)	
**Tumor type**	Primary Recurrence (indeterminate, likely, definite) - Type: local, nodal, peritoneal, other
**Location**	Central compartment Anterior compartment Posterior compartment Lateral compartment Detailed prose description of location: ….
**Size**	Longest dimension: … cm (Other dimensions: … x … cm)
**II—(Potentially) involved organs and structures**	
**For each organ/structure describe certainty of involvement**	…. Margin Focal contact Broad-based contact Frank invasion
**BONVUE acronym** **(which structures to report)**	Bones Organs Nerves Vessels Extra tumor sites
**Reporting tips for specific organ/structure involvement**	**Vessels:** describe longitudinal + radial extent of invasion, associated luminal narrowing, occlusion and/or deformity, and presence of thrombus or collaterals **Ureters:** describe longitudinal + radial extent of invasion, presence + degree of upstream dilatation, signs of impaired kidney function (hydronephrosis, cortical thinning decreased enhancement) **Sacral nerves:** describe which nerve roots are involved, and the distance (free margin) between the tumor and closest non-involved nerve root **Bones**: discern superficial and/or deep bone invasion; in case of sacral bone invasion describe which vertebra are involved **Anal canal & pelvic floor**: describe which layers (internal sphincter, intersphincteric plane, external sphincter) are involved, and craniocaudal extent of invasion
**III—Additional tumor sites**	
**Associated lymph node metastases**	No Yes: … (including specified description of pelvic side wall nodes)
**Extra-pelvic disease sites**	No Yes: ….
**IV—Other**	
**Anatomic variants**	eg, aberrant vessels
**Post-treatment changes**	From previous surgery or radiotherapy
**V—Restaging (only applicable if imaging is performed after neoadjuvant treatment)**	
**Estimated degree of response**	Progression Stable disease Partial response Very good (including possible complete) response
**Reduction in size (longest diameter) compared to prior study**	From …. cm to … cm
